# Body mass but not vitamin D status is associated with bone mineral content and density in young school children in northern Sweden

**DOI:** 10.3402/fnr.v60.30045

**Published:** 2016-03-03

**Authors:** Frida K. Videhult, Inger Öhlund, Olle Hernell, Christina E. West

**Affiliations:** Pediatrics, Department of Clinical Sciences, Faculty of Medicine, Umeå University, Umeå, Sweden

**Keywords:** dual energy X-ray absorptiometry (DXA), serum 25-hydroxyvitamin D, season, latitude, diet, calcium

## Abstract

**Background:**

High latitude of residence where sun exposure is limited affects vitamin D status. Although vitamin D levels have been associated with poor bone health, cut-off values for optimising bone health are yet to be decided.

**Objective:**

To assess vitamin D intake and status among young school children living at latitude 63–64 °N, in northern Sweden and to examine the association between vitamin D status and bone mineral content (BMC) and bone mineral density (BMD).

**Design:**

In a cross-sectional study, diet was assessed by a 4-day food diary and a food frequency questionnaire in 8- to 9-year-old children (*n*=120). Energy, vitamin D, and calcium intakes were calculated. Physical activity was assessed using a pedometer for 7 days. Serum 25-hydroxyvitamin D (S-25[OH]D) levels were analysed by high-pressure liquid chromatography-atmospheric pressure chemical ionisation-mass spectrometry (*n*=113). BMC and BMD were assessed by dual energy X-ray absorptiometry scan. Height and weight were measured by standard procedures and BMI *z*-score was calculated using WHO AnthroPlus programme.

**Results:**

The majority of children, 91%, did not reach the recommended vitamin D intake of 7.5 µg/day and 50% had insufficient S-25[OH]D levels defined as <50 nmol/l. The highest concentrations of S-25[OH]D were observed during the summer months (*p*=0.01). Body mass (*p*<0.01) but not S-25[OH]D was associated with measures of BMC and BMD. Furthermore, boys had higher total BMC (*p*=0.01), total body less head BMC (*p*=0.02), fat free mass (*p*<0.01), and a higher degree of physical activity (*p*=0.01) compared to girls.

**Conclusions:**

Body mass was related to BMC and BMD measures in a population of prepubertal school children living at high latitudes in Sweden. Despite insufficient S-25[OH]D levels and low vitamin D intake, this did not appear to affect bone parameters. Prospective studies with repeated assessment of vitamin D status are needed to examine cut-off values for optimising bone health.

The interest in vitamin D status and its health effects in young populations has increased in recent years (1). Studies in children and adolescents are rapidly increasing worldwide and available data indicate low vitamin D status across all age groups (2). Vitamin D status is important for optimising bone health at an early age (3); however, lower vitamin D status has been associated with a number of disorders, such as obesity, cardiovascular, and metabolic disease (4). Serum or plasma 25-hydroxyvitamin D (25[OH]D) is the most reliable biomarker because it mirrors both the amount of vitamin D produced in the skin by UVB-radiation following sunlight exposure and the intake from dietary sources (3). Consequently, to estimate the contribution of dietary vitamin D intake to the 25[OH]D level is difficult (5). Latitude of residence appears to be a predictor for seasonal variation in vitamin D status (5), although inadequate vitamin D status is reported also in low latitude countries (2). Cut-off values for suboptimal vitamin D status are not clearly defined in the pediatric population (1, 3). Using bone health as an outcome, some advocate that S-25[OH]D levels of ≥50 nmol/L is adequate in healthy children (1, 6), but that levels of >75 nmol/L should be strived for in populations at risk for fractures, that is, children with obesity, lighter skin, and those with conditions associated with reduced bone mass (3).

In 2011, the Institute of Medicine (IOM), endorsed by the American Academy of Pediatrics, updated the dietary reference intakes for vitamin D to 600 IU/day (15 µg/day) for children 4–13 years of age. The corresponding recommendation for calcium was revised to 1,000 mg/day for children 4–8 years of age and 1,300 mg/day for children in the age category 9–13 years (3). The recommendation for daily intake of vitamin D in Sweden was recently increased from 7.5 to 10 µg/day for vitamin D in children aged 6–9 years (7), whereas the recommendation for calcium remains the same, that is, 700 mg/day.

Bone is an active ‘dynamic’ tissue, where old bone is replaced with new bone throughout the life span (8). During early childhood to late adolescence skeletal mass is successively accumulated and at around 18 years of age 90–95% of bone accrual appears completed (3, 8). Many factors influence bone turnover, for example, hormonal status, smoking, diet, and physical activity. However, up to 80% of bone mineral density (BMD) is genetically determined (8), leaving 20% to be influenced by environmental factors such as exercise and dietary intake of calcium. Low vitamin D levels directly affect the skeletal mineralisation because of reduced efficiency of intestinal calcium absorption (8).

The global obesity epidemic has also spurred interest in vitamin D status (9), as low levels of 25[OH]D have been reported in obese individuals. However, most clinical studies have so far been on adults. The exact relationship between vitamin D status and obesity is yet to be decided and results are conflicting regarding effects of obesity on bone health in children and adolescents (10).

We have previously shown that vitamin D status is inadequate and that vitamin D insufficiency defined as S-25[OH]D <50 nmol/L is common in preschool children (11) and adolescents (12) living in northern Sweden. Therefore, we hypothesised that vitamin D status in prepubertal 8- to 9-year-old children living at latitudes 63–64 °N would be insufficient and that low vitamin D status would be associated with lower bone mineral content (BMC) and BMD measured by dual energy X-ray absorptiometry (DXA).

## Method and materials

### Participants

This is a cross-sectional study including 8- to 9-year-old children participating in a follow-up of a double-blind, randomised, placebo-controlled intervention trial (registered at www.clinicaltrials.gov [NCT 00894816]) and reported in detail elsewhere (13). Briefly, families with a healthy, newborn child in the area of Umeå city, Västerbotten County, Sweden, were invited to participate in this allergy prevention study through well-baby clinics and advertisements during 2000 to 2003. Of 179 included infants, 89 infants were randomised to daily intake of cereals with *Lactobacillus paracasei* ssp. *paracasei* F19 (LF19) and 90 infants to daily intake of cereals without LF19 from 4 to 13 months of age. In total, 171 infants completed the intervention. At age 8–9 years, 120 children participated in a follow-up study from 2009 to 2011. All participants were of Caucasian origin, living in Västerbotten County, latitude 63–64 °N. Written and oral information was provided before enrolment and a written consent was signed by the parents. Approval of the trial was obtained from the local ethical review board in Umeå.

### Biochemistry

Venous blood samples were collected after overnight fasting using Vacutainer^®^ tubes (Becton Dickinson, Plymouth, UK) from January to December (2009–2011). However, July is not included as no blood sampling was done because of the holiday season. After centrifugation within an hour, serum was frozen at –20°C and then stored at –70°C until analysis. S-25[OH]D was analysed according to high-pressure liquid chromatography-atmospheric pressure chemical ionisation-mass spectrometry (HPLC-APCI-MS) (Vitas, Oslo, Norway) (14).

### Anthropometric measurements and body composition

BMD and BMC were evaluated by using DXA (Lunar prodigy whole-body scanner GE Medical Systems, Madison, WI, USA) with the child in supine position. Manufacturer-supplied software (versions 8.70 and 13.31) was used; difference in versions did not affect the outcome because the reference population had not changed (NHANES/USA whole body reference population (v101)). In the present analyses BMC is reported as total BMC and total body less head (TBLH) BMC. For version 8.70 this is calculated as total BMC minus head BMC; in the later version TBLH BMC is provided by the software. Of the 120 children included in the study, 21 were measured using the latter version. The procedure of the measurements on body composition has previously been described (15). Briefly, height and weight were measured using standard procedures and from these measurements BMI *z*-score was calculated using the WHO AnthroPlus programme (15). From the DXA scans, fat free mass (FFM) was calculated as; BMC plus lean mass (16). Furthermore, fat mass index (FMI) (fat mass (kg)/m^2^) and fat-free mass index (FFMI) (fat-free mass (kg)/m^2^) were calculated (17). The same research nurse performed all scans using standard clinical procedures.

### Vitamin D intake and physical activity

Dietary intake of vitamin D and calcium was assessed by a 4-day food record which recorded type and quantity of food items consumed by the child. In addition to the food record, families also filled in a short-food frequency questionnaire (FFQ). Questions on consumption of dairy products and the child's dietary pattern, that is, *omnivore*, *lacto vegetarian*, *vegan*, or *other*, were analysed. The daily intakes of vitamin D and calcium were calculated for each child in a dietary analysis programme Dietist XP version 3.2 2011-03-10 (Kost och Näringsdata AB, Stockholm). Dietist XP was based on the Swedish Food composition database version 2011-02-14. As mentioned, the recommended intake of vitamin D for this age group at the time of the data collection was 7.5 µg/day. Here, S-25[OH]D levels >50 nmol/l were regarded as sufficient (1, 6). Physical activity was reported as mean number of steps following a 7-day registration (15).

### Statistics

Descriptive data are displayed as mean (SD) for normally distributed variables and displayed as median (25th, 75th percentile) for non-normally distributed variables. Categorical variables are presented as frequencies (%). A student's *t* test was performed for differences in BMC and BMD, body mass measurements, physical activity, and S-25[OH]D levels between girls and boys. We categorised the children according to the quarter of blood sampling, that is, first quarter (Q1), January through March; second quarter (Q2), April through June; third quarter (Q3), July through September (no child was tested during July because of summer holidays); and fourth quarter (Q4), October through December. A Kruskal–Wallis test was performed to examine whether there was a difference in S-25[OH]D levels according to the quarter of blood sampling. Pearson's correlation was conducted for relationships between bone measures from DXA, BMI *z*-score, S-25[OH]D, mean kcal, vitamin D, and calcium intake. A standard multiple linear regression with vitamin D levels as a continuous variable divided by quarters of the year, that is, Q1–Q4 according to the time of blood sampling (as described above), sex, and physical activity as independent variables and BMD, BMC, DXA (i.e. FMI and FFMI), and BMI *z*-score as dependent variables, was performed.

*p*-Value of <0.05 was set as statistically significant. Data were analysed with SPSS software version 21.0 (SPSS Institute, Inc., Chicago).

## Results

### Characteristics of the study population

Descriptive characteristics of the children (*n*=120) and their parents are presented in Table 1. This study was part of a follow-up of an allergy prevention study, and 37 children were diagnosed with allergic disease (eczema, food allergy, and/or respiratory allergy) at 8–9 years of age (18). Other chronic disease diagnoses were also queried and included: celiac disease (*n*=1), lactose intolerance (*n*=1), congenital heart disease (*n*=1), and enuresis (*n*=1). Some children had two or more diagnoses.

**Table 1:** Descriptive characteristics of the study population (*n*=120)

	All (*n*=120)
Boys (%)	43
Age at follow-up (year)	8.8 (2.0)
Overweight/obese (%)	20
Maternal university education (%)	68
Paternal university education (%), *n*=119	49
Maternal smoking (%)	8
Paternal smoking (%), *n*=117	6
Overweight/obese mothers (%), *n*=117	35
Overweight/obese fathers (%), *n*=112	55
Maternal BMI, *n*=117	24.3 (3.5)
Paternal BMI, *n*=112	26.1 (3.5)

BMI, body mass index. Normally distributed data, presented as mean (SD).

### Children reported a low dietary vitamin D intake

None of the children participating in the study excluded meat or dairy products completely during the data collection, although 8% reported that they never drank milk. Overall, the mean (SD) frequency of daily milk consumption was 2.4 (1.2) times per day. The majority (93%) used vitamin D fortified table spread. Fifteen per cent of the children reported vitamin D and calcium containing supplement use. There was a wide variation in type of supplement and usage per day, week, or month (data not shown). Based on the food records, the mean (SD) intake of vitamin D was 4.7 (2.0) µg, corresponding to 63% of the daily recommended intake in Sweden at the time of the study and 91% had a lower intake compared to recommendations. Mean (SD) calcium intake was 936 (331) mg. Twenty-five per cent of the children reported calcium intakes below the recommended daily intake (RDA), that is, 700 mg.

### Every second child had insufficient levels of S-25[OH]D

**Fig. 1:**
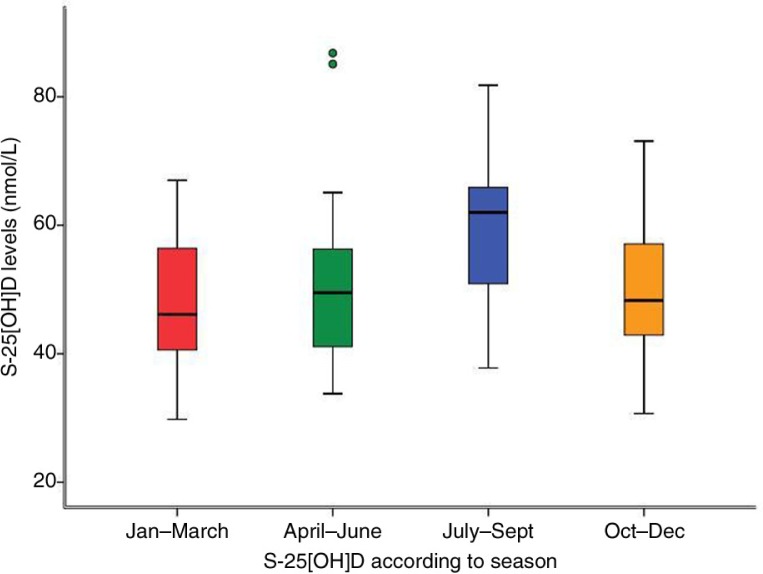
Box plot of S-25[OH]D during the four different quarters of the year. The values represent the median (horizontal lines), interquartile range (box), and range of values (whiskers). The children tested during the third quarter (July–September) had the highest measured levels of S-25[OH]D (Md=62 nmol/L), the other groups (January–March, April–June, and October–December) had median levels of 46, 50, and 48 nmol/L, respectively.

In total, 50% of the children had insufficient (<50 nmol/L) and only 5% (*n*=6) of the children had S-25[OH]D levels >75 nmol/L. One single child had S-25[OH]D levels <30 nmol/L. S-25[OH]D levels did not differ between girls and boys (Table 3). As expected, the highest median S-25[OH]D levels (*p*=0.01) were observed during summer, that is, in July, August, and September (Fig. 1) as compared to the other seasons.

### BMC and BMD were adequate in most children

We also assessed BMD, BMC, and body mass in the study population (Table 2). Eighty-two per cent of the children had a BMD *z*-score ≥0.0. Mean (SD) total BMC was 1,147.8 (119.2) g and mean (SD) TBLH BMC was 811.5 (175.1) g. Next, we assessed the relationship between BMC, BMD, body mass, and S-25[OH]D levels. In the multiple linear regression model, sex had an effect on total BMC (*β*=0.29, *p*=0.01, *95%* CI 35.0–196.8), TBLH BMC (*β*=0.26, *p*=0.01, *95%* CI 19.4–162.7), FFMI (*β*=.44, *p<*0.01, *95%* CI 0.51–1.29), and BMI *z*-score (*β*=0.22, *p*=0.04, *95%* CI 0.02–0.96) with higher levels among boys. S-25[OH]D level according to quarter of blood sampling and physical activity did not show any significant correlation with measures of BMC, BMD, and body mass (data not shown).

**Table 2:** Measurements of body mass and bone parameters

	All (*n*=120)
BMI *z*-score	0.4 (1.1)
FMI*, *n*=117	3.7 (2.8, 5.1)
FFMI, *n*=117	12.8 (1.0)
BMD *z*-score	0.6 (0.7)
Total BMD (g/cm^2^), *n*=117	0.89 (0.05)
BMD pelvis (g/cm^2^), *n*=117	0.80 (0.06)
BMD LS (g/cm^2^), *n*=117	0.70 (0.05)
Total BMC (g), *n*=117	1147.8 (119.2)
TBLH BMC (g), *n*=117	811.5 (175.1)
BMC pelvis (g), *n*=117	118.0 (25.1)
BMC LS (g), *n*=117	90.8 (18.7)

BMI, body mass index; FMI, fat mass index; FFMI, fat free mass index; BMD, bone mineral density; LS, lumbar spine; BMC, bone mineral content; TBLH, total body less head. Normally distributed data, presented as mean (SD).

* non-normally distributed data, presented as median (25th, 75th percentile).

### Boys were more physically active with higher BMC and FFM

Total BMC, TBLH BMC, FFM, and physical activity were statistically significantly higher among boys compared to girls (Table 3). As shown in Table 3, there were no observed sex differences in the other measurements of BMC, BMD, body mass, or S-25[OH]D levels.

**Table 3:** Measures of bone mineral density, bone mineral content, dietary intakes and physical activity in boys and girls at 8–9 years of age

	Boys (*n*=52)	Girls (*n*=68)	*p*
BMD pelvis (g/cm^2^)†	0.8 (0.1)	0.8 (0.1) (*n*=65)	0.54
BMD LS (g/cm^2^)†	0.7 (0.1)	0.7 (0.1) (*n*=65)	0.70
Total BMD (g/cm^2^)†	0.9 (0.1)	0.9 (0.1) (*n*=65)	0.10
BMD *z*-score†	0.6 (0.7)	0.5 (0.6) (*n*=65)	0.48
BMC pelvis (g)†	121.5 (26.6)	115.1 (23.7) (*n*=65)	0.17
BMC LS (g)†	91.1 (20.2)	90.6 (17.6) (*n*=65)	0.90
Total BMC (g)†	1200.9 (211.3)	1104.3 (179.1) (*n*=65)	**0.01**
TBLH BMC (g)†	853.3 (187.4)	778.0 (158.2) (*n*=65)	**0.02**
BMI *z*-score	0.7 (1.2)	0.2 (1.1)	0.05
FFM (kg)†	25.8 (3.9)	22.9 (2.7) (*n*=65)	**<0.01**
FM (kg)†	8.1 (5.0)	8.2 (4.5) (*n*=65)	0.98
Total body fat (%)†	23	26 (*n*=65)	0.10
Energy intake (kcal)†	1780 (342) (*n*=50)	1667 (381) (*n*=63)	0.10
Vitamin D intake (µg)†	5.1 (2.2) (*n*=50)	4.4 (1.8) (*n*=63)	0.06
Calcium intake (mg)†	967 (382) (*n*=50)	905 (283) (*n*=63)	0.27
Physical activity (no. steps)†	9210 (2785) (*n*=50)	7275 (2962) (*n*=60)	**0.01**
S-25[OH]D (nmol/L)†	51.5 (12.5) *(n*=50)	51.6 (11.8) (*n*=63)	0.96

BMD, bone mineral density; LS, lumbar spine; BMC, bone mineral content; TBLH, total body less head; BMI, body mass index; FFM, fat free mass; FM, fat mass. Data presented as mean (SD), *p* values based on student's *t* test. Bold font indicates statistical significance.

† Data not available from all children, numbers included in brackets.

### Body mass, energy, and calcium intake affected BMC and BMD

BMI *z*-score, FFMI, and FMI showed intermediate to strong positive correlations with BMC and BMD variables, ranging from *r*=0.341 to *r*=0.705, all *p*<0.01 (Table 4). These significances remained when analysing each sex separately. Next, we analysed energy, calcium, and vitamin D intake and bone measures. As shown in Table 5, mean energy intake was positively correlated with all measures of BMC and BMD with the exception of lumbar spine (LS) measurements. Furthermore, calcium intake correlated positively with pelvis BMD, total BMD, and BMD *z*-score, but not with LS BMD and BMC measurements. Vitamin D intake showed a negative correlation with FMI but with no other reported variable. There were no significant correlations between S-25[OH]D and the measured variables or with vitamin D and calcium intake (data not shown). However, calcium and vitamin D intake displayed a strong positive correlation (*r*=0.59, *p*<0.01) as well as calcium and mean energy intake (*r*=0.62, *p*<0.01).

**Table 4:** Bone parameters were strongly correlated to body composition as assessed by DXA

	BMD pelvis (g/cm^2^)	BMD LS (g/cm^2^)	Total BMD (g/cm^2^)	BMD *z*-score	BMC pelvis (g)	BMC LS (g)	Total BMC (g)	TBLH BMC (g)
BMI *z*-score	.49* (<**0.01**)	.47* (<**0.01**)	.48* (<**0.01**)	.4* (<**0.01**)	.53* (<**0.01**)	.71* (<**0.01**)	.68* (<**0.01**)	.70* (<**0.01**)
FFMI	.47* (<**0.01**)	.40* (<**0.01**)	.52* (<**0.01**)	.46* (<**0.01**)	.55* (<**0.01**)	.47* (<**0.01**)	.61* (<**0.01**)	.60* (<**0.01**)
FMI	.39* (<**0.01**)	.39* (<**0.01**)	.36* (<**0.01**)	.34* (<**0.01**)	.40* (<**0.01**)	.69* (<**0.01**)	.59 (<**0.01**)	.62* (<**0.01**)

BMD, bone mineral density; LS, lumbar spine; BMC, bone mineral content; TBLH, total body less head; BMI, body mass index; FFMI, fat free mass index; FMI, fat mass index.

* Values show correlation coefficients (*r*), values in parentheses are the corresponding *p* values. Bold font indicates statistical significance. Correlation significant at the 0.01 level (two-tailed).

**Table 5:** Pearson's correlation between S-25(OH)D status, mean energy intake, vitamin D and calcium intake, bone mineral density, bone mineral content and body composition as assessed by DXA-scan

	BMD pelvis (g/cm^2^)	BMD LS (g/cm^2^)	Total BMD (g/cm^2^)	BMD *z*-score	BMC pelvis (g)	BMC LS (g)	Total BMC (g)	TBLH BMC (g)	BMI z-score	FFMI	FMI
S-25(OH)D (nmol/L)	−.12 (0.20)	−.06 (0.55)	−.03 (0.76)	−.06 (0.50)	−.13 (0.16)	−.11 (0.25)	−.11 (0.24)	−.11 (0.26)	−.05 (0.59)	.07 (0.47)	−.11 (0.23)
Mean energy intake (kcal)	.23* (**0.02**)	.15 (0.11)	.26** (**0.01**)	.25** (**0.01**)	.22* (**0.02**)	.12 (0.21)	.22* (**0.02**)	.20* (**0.03**)	.03 (0.73)	.26** (**0.01**)	−.11 (0.26)
Mean vitamin D (µg)	.11 (0.24)	.02 (0.84)	.16 (0.09)	.13 (0.17)	.06 (0.52)	−.06 (0.51)	.02 (0.82)	−.00 (0.98)	−.12 (0.20)	.15 (0.12)	−.20* (**0.04**)
Mean calcium (mg)	.20* (**0.03**)	.15 (0.12)	.20* (**0.04**)	.21* (**0.03**)	.15 (0.11)	.02 (0.83)	.12 (0.22)	.10 (0.31)	.00 (0.99)	.16 (0.09)	−.09 (0.32)

BMD, bone mineral density; LS, lumbar spine; BMC, bone mineral content; TBLH, total body less head; BMI, body mass index; FFMI, fat free mass index; FMI, fat mass index. Values show correlation coefficients (*r*), values in parentheses are the corresponding *p* values. Bold font indicates statistical significance.

* Correlation significant at the 0.05 level (two-tailed).

** Correlation significant at the 0.01 level (two-tailed).

## Discussion

Vitamin D intake in the present study population was low and did not meet the national recommendations in 91% of the children. Half of the children presented insufficient S-25[OH]D levels (<50 nmol/L) which confirm the findings from our previous studies in this high latitude region (11, 12). Still, although half of the children had insufficient S-25[OH]D levels, this did not influence BMC and BMD measures. As only one child had S-25[OH]D levels <30 nmol/L, our results could be consistent with guidelines from IOM stating that children are at risk of poor bone health at S-25[OH]D levels <30 nmol/L. However, as some may still be at risk in the range of 30–50 nmol/L, S-25[OH]D >50 nmol/L should generally be the population target (6).

Another finding of this study was that body mass, fat mass, and FFM, were strongly associated with BMC and BMD. Whether the impact of lean mass or fat mass is most beneficial for bone status during growth is a matter of discussion. Lean mass has been shown to be the most important predictor for bone growth in children (19) and adolescents (20). Here we found almost equally strong correlations between FFMI and FMI, indicating that body mass regardless of composition is of relevance for BMC and BMD in prepubertal children. It has been speculated that fat mass becomes of importance for increased bone mass only after puberty and that lean mass is important before puberty (10). However, in this cohort none of the children had reached puberty according to Tanner criteria. Because of the adverse health effects associated with obesity, an important question raised but not addressed in the present study is how the degree of obesity interferes with bone health. Furthermore, it is important to note that obese children not only have more fat mass but also more lean mass.

Little is known about cut-off values indicating adequate bone health in a healthy population, which makes inferences difficult. Low BMD *z*-score has, however, been defined as less than or equal to –2.0 (21). Our results of BMD *z*-score (0.6) are in accordance with a Finnish study in adolescents where median whole body BMD *z*-score was 0.0 (22). However, prepubertal BMD scan has low predictive ability for assessment of people at risk of low peak bone mass (23). Because DXA measures BMD area and not volume, true BMD might be overestimated in large bones and underestimated in small bones in growing individuals, which make some researchers favour BMC (10). BMC is preferably measured as TBLH BMC during growth because of changes in relative contribution of the head to total BMC and the importance of postcranial skeleton in fracture risk assessment (24). Collectively, the children in our study appear to have good bone health measured as BMC and BMD (21, 24).

In contrast to the very low reported vitamin D intakes in the present study, approximately 75% of the children reported calcium intakes above the recommended daily intake (RDA) of 700 mg Ca/day. This could be explained by a high intake of dairy products or the use of calcium supplements. A Cochrane review from 2010 on supplementation with vitamin D for improvement of BMD in children concluded that supplementation in children with vitamin D deficiency might be of clinical importance (25), but there was no support for supplementation in healthy children with normal vitamin D levels. We could not see any correlation with S-25[OH]D levels or vitamin D intake and BMC or BMD measures or body mass with the exception of vitamin D intake and FMI, which displayed a negative association. Plausible explanations are that only one child had S-25[OH]D <30 nmol/L, which is suggested to be a cut-off level for risk of poor bone health, that is, rickets and osteomalacia (6) and that there is a seasonal variation in vitamin D status (26). Here, we measured the S-25[OH]D levels on one isolated occasion in each child which fails to capture the overall individual shifts of vitamin D throughout the year as a result of sun exposure and dietary intake. Early nutrition is important for achieving an optimal peak bone mass and infant dietary patterns with high intake of dairy, cheese, and eggs are positively associated with bone development in childhood (27). Our results show that mean energy intake as well as intake of calcium was positively associated with several BMD and BMC variables.

Boys had higher levels of total BMC, TBLH BMC, FFM, and physical activity, compared with girls, which is in accordance with others (20). FFM and physical activity are known to increase bone mass (3). In Danish 9-year-old children, inexpensive measurements, such as height and BMI, were positive predictors of bone accrual in childhood (28). However, these measurements were insensitive to sex differences, which can be detected by more sophisticated measures such as DXA. Here, we report equally strong correlations between BMI *z*-score, the selected DXA variables, and BMC and BMD, independently of sex.

Study strengths are the use of DXA as a measure of bone parameters, which is the clinically most used technique to estimate bone properties and ideal for paediatric use because of its rapid scan time and low radiation exposure (24) and the use of gold standard HPLC-APCI-MS for assessment of S-25[OH]D levels (14). A possible study limitation is the lack of blood samples for analysis of alkaline phosphatase and parathyroid hormone as well as the lack of longitudinal data. Another limitation might be the representativeness of the study population, as these children were included as a part of a follow-up of an intervention trial in infancy. Although the trial approached the general population, families with allergic heredity were more prone to participate (18). Reassessment of approximately 70% might also have introduced some biases to the results. Thus the sample selection might not be fully representative of prepubertal children in northern Sweden. Although we combined 4-day food records to FFQ to cover dietary intakes, it is known that there are limitations to this approach (29, 30). This, and the lack of registration of sun exposure, might explain why there was no correlation between the dietary vitamin D intake and S-25[OH]D levels in this population.

## Conclusions

In this study of prepubertal school children aged 8–9 years, we found that both fat mass and FFM correlate with measures of bone health, that is, BMC and BMD. The low vitamin D intake and low levels of S-25[OH]D did not seem to affect BMC and BMD at this stage, nevertheless, potential long-term adverse effects cannot be excluded. Future studies should focus on prospective repeated measurements of vitamin D status and bone health through childhood and adolescence, especially in at-risk groups, that is, those with underlying medical conditions, dark skin, a deviating diet, or in the obese.
